# Understanding the pneumococcus: transmission and evolution

**DOI:** 10.3389/fcimb.2013.00007

**Published:** 2013-03-07

**Authors:** Eric S. Donkor

**Affiliations:** Department of Microbiology, University of Ghana Medical SchoolAccra, Ghana

**Keywords:** pneumococcus, evolution, transmission, carriage, recombination

## Abstract

*Streptococcus pneumoniae* is part of the normal bacterial flora of the narsopharynx, but is also associated with several invasive and non-invasive diseases. Recently, there has been a plethora of research information on the pneumococcus, however, there are few comprehensive review papers discussing the research information. This paper provides a review of the pneumococcus in two vital areas related to its biology including transmission and evolution. Transmission of the pneumococcus is a highly efficient process that usually occurs through respiratory droplets from asymptomatic carriers. Following acquisition, the pneumococcus may only establish in the nasopharynx of the new host, or further progress to sites such as the lungs and cause disease. Pneumococcus transmission risk factors, as well as factors involved in its translocation from the nasophyarnx to diseases sites are still not fully understood. Pneumococcal evolution is dominated by recombination. The recombinational events usually involve genetic exchange with streptococci of the mitis group and some pneumococci are thought to exhibit hyper-recombination.

## Introduction

The pneumococcus is one of the most virulent human pathogens and causes a wide range of infections, including invasive and non-invasive diseases. There are about one million new pneumococcal infections every year, majority of which occur among children <5 years, and the organism is responsible for 10–20% of all deaths in this age group (O'Brien et al., 2009). Until about two decade ago, little was known about *Streptococcus pneumoniae*. However, since this time there has been a plethora of research information contributing significantly to our understanding about this very important human pathogen. Unfortunately, there are few recent comprehensive review papers discussing the plethora of research information about the pneumococcus. This paper provides a review of the pneumococcus in two vital areas related to its biology including transmission and evolution.

## *S. pneumoniae* carriage and diseases

*S. pneumoniae* is part of the normal bacterial flora of the upper respiratory tract of humans, and is mainly found in the nasopharynx. Carriage of the organism is more prevalent in children than adults; the colonization rate rises from birth until it peaks around the age of 1–2 years, and thereafter an age related decline is observed (Lloyd-Evans et al., 1996; Hussain et al., 2005). *S. pneumoniae* carriage normally lasts for a couple of weeks, and duration periods of more than 30 weeks are observed (Sleeman et al., 2008). A seasonal carriage trend has also been described, with peak rates occurring during January–March (Gray et al., 1980). Children acquire several different strains over time, and less immunogenic serotypes tend to be carried in the narsopharynx for a much longer period of time than the more immunogenic serotypes (Rosen et al., 1984; Obaro and Adegbola, 2002).

In developing countries, carriage rates are relatively higher especially, in children. In the Gambia, Lloyd-Evans et al. (1996) reported a carriage rate of 80% among children under five years of age, and a lower rate of 20% in adults. In a study by Gratten et al. (1986), it was found that 60% of infants in Papua New Guinea acquired *S. pneumoniae* already during the neonatal period, and all infants were colonized within the first 3 months of life (Gratten et al., 1986). Similar high *S. pneumoniae* carriage rates in the developing world have been reported in several other countries including Zambia (Frederiksen and Henrichsen, 1988), Pakistan (Mastro et al., 1993), The Philippines (Lankinen et al., 1994), Papua New Guinea (Gratten et al., 1986), and Bangladesh (Granat et al., 2007). In the developed world, *S. pneumoniae* carriage appears to be lower than rates found in the developing world. Aniasson et al. (1992) reported that in Sweden, only 12% of infants were colonized with *S. pneumoniae* at 3 months, 30% at 7 months, and 32% at 12–18 months. In the UK, a longitudinal study by Goldblatt et al. (2005) showed an overall prevalence rate of 25%, with carriage rates of 52 and 8% in children under 2 years and adults over 18 years of age, respectively. Labout et al. (2008) studied *S. pneumoniae* carriage in infants in the Netherlands and observed carriage rates of 8.3% at age 1.5 months, 31.3% at 6 months, and 44.5% at 14 months. The high pneumococcal carriage rates in the developing world appear to provide more opportunities for multiple carriage, as relatively higher multiple carriage rates have also been reported in the developing countries compared to the developing world (Gratten et al., 1994; Obaro et al., 1996; Brugger et al., 2010).

The major diseases caused by *S. pneumoniae* include pneumonia, meningitis, septicaemia, and otitis media. There are two types of pneumonia, namely, bronchial pneumonia and lobar pneumonia, and *S. pneumoniae* is a major cause of both types. In an infection of pneumonia, *S. pneumoniae* stimulates the immune system and causes migration of white blood cells to the lungs. The interaction of white blood cells, proliferating bacteria and excessive fluid define the presence of pneumonia (Tuomanen et al., 1995), which can be detected by a chest X-ray. Bacteraemia and septicaemia can occur in 20–30% of cases with pneumococcal pneumonia (Musher, 2004). *S. pneumoniae* meningitis perhaps has the highest case fatality rate (~40%) among the various pneumococcal infections and up to 50% of survivors suffer from debilitating sequalae such as mental retardation and motor deficiency (Bohr et al., 1984; Leimkugel et al., 2005). The clinical presentations of meningitis caused by *S. pneumoniae* are similar to other bacterial causes of acute meningitis and include severe headache, photophobia, neck stiffness, and fever. Otitis media is the most common of the pneumococcal diseases. The condition is an inflammation of the middle ear and up to 50% of the cases are caused by *S. pneumoniae* (Musher, 2004). The major clinical signs of the infection include limited mobility and enlarged tympanic membrane. It is reported that successful treatment with antibiotics can still lead to recurrent otitis media due to proximity of the middle ear to the nasopharynx where *S. pneumoniae* resides (Libson et al., 2005). Other *S. pneumoniae* diseases include conjunctivitis, acute tracheobronchitis, endometritis, peritonitis, endocarditis, arthritis, and osteomyelitis. However, these infections are relatively uncommon.

## Transmission of *S. pneumoniae*

As humans are the main host for *S. pneumoniae*, successful transmission of *S. pneumoniae* among humans is crucial for survival of the organism, and without this the pneumococcus is likely to be eliminated. Transmission of *S. pneumoniae* occurs through respiratory droplets from people with pneumococcal disease or more commonly healthy individuals who carry the organism in the nasopharynx (Bogaert et al., 2004; Sleeman et al., 2005). Little is known about the risk factors of pneumococcal transmission, though certain risk factors including number of siblings and visits to general practitioners for mild upper respiratory disease have been identified (Sleeman et al., 2005). Additionally, higher rates of pneumococcal transmission are known to occur at certain sites including day care centers, military camps, and prisons (Givon-Lavi et al., 2002; Bogaert et al., 2004). Givon-Lavi et al. (2002) compared pneumococcal isolates recovered from children in day care centers with isolates recovered from younger siblings not attending day care by pulsed-field gel electrophoresis. This showed a high level of genetic similarity among isolates from the specific day care center the older sibling was attending and those isolated from younger siblings, which indicate that pneumococcal transmission may be a highly efficient process.

Following acquisition, the pneumococcus may establish in the nasopharynx of the new host, and in most cases this leads to asymptomatic colonization (Sleeman et al., 2008). However, occasionally, the newly acquired pneumococcus moves from the nasopharynx to other parts of the human host such as the lungs where it evades the host defence mechanisms and causes disease (Bogaert et al., 2004). Because asymptomatic carriers far exceed symptomatic individuals, most of the links in the transmission chain of person-to-person are not visible. In contrast, a respiratory disease such as measles is also transmitted person-to-person through the same route as pneumococcus but asymptomatic colonization does not happen, and each link in the transmission chain is evident as disease (Mrozek-Budzyn, 2010). There is evidence that the risk for progression of pneumococcus from asymptomatic colonization to disease seems to be greatest soon after acquisition and a complex interplay of factors are involved (Sleeman et al., 2005). Colonizing pneumococci strains may elicit an immune response that may eliminate them (Obaro and Adegbola, 2002). Additionally, the composition of the microflora of the nasopharynx, which is thought to contain more than 700 diverse species, may support or hinder colonization and invasion by symbiosis and/or competition (Aniasson et al., 1992; Harputluoglu et al., 2005). There are significant differences in the attack rate of different serotypes, where attack rates refer to the incidence of invasive pneumococcal disease per the incidence of pneumococcal acquisition. Pneumococcal serotypes such as 1, 4, 5, and 9A have high attack rates, while serotypes such as 9N, 16F, 20, and 38 have low attack rates; generally, attack rates are higher for serotypes which are carried for short time periods (Sleeman et al., 2005). Phase variation where pneumococcal variants have the same serotype but vary from opaque to transparent colonies, is thought to be important in the progression of *S. pneumoniae* from carriage to invasive disease (Weiser et al., 1994; Arai et al., 2011). This is because the opaque form has been commonly isolated from patient samples, while the transparent form is adapted to colonization of the nasopharynx. According to Ring et al. (1998), phase variation of the transparent type increases pneumococcal invasion into human brain microvascular endothelial cells as much as six-fold.

## Evolution of *S. pneumoniae*

Evolution of the pneumococcal population is known to be dominated by recombination. Using MLST data, Feil et al. (2000) demonstrated that the rate of recombination in *S. pneumoniae* was 10 times higher than the rate of mutation, while for *Neisseria meningitidis*, the rate of mutation was five times higher than the rate of mutation. The high rate of pneumococcal recombination has recently been illustrated by the whole genome sequencing of 240 strains of a single lineage, ST 81 (Croucher et al., 2011a). This showed that over 700 recombinational events had occurred in this pneumococcal lineage and 74% of the genome length had undergone recombination in at least one isolate. Currently, it is unknown whether this observation holds for all pneumococcal lineages or whether certain geographical settings could lead to yet more recombination. In contrast to the frequent pneumococcal recombination, mutation is known to be more important than recombination in evolution of clonal bacteria such as *Staphylococcus aureus* (Feil et al., 2003). The high rates of pneumococcal recombination may be attributed to the relatively high density of repeat elements in the genome that may facilitate incorporation of foreign DNA into the *S. pneumoniae* chromosome and contribute to rearranging its structure. Aras et al. (2003) analysed the density of repeats for 51 prokaryotic genomes, and observed that *S. pneumoniae* had the greatest density of 1 every 500 bp. Until recently, two types of such repeat elements including BOX and RUPS elements were known to occur in the *S. pneumoniae* genome. However, a study by Croucher et al. (2011b) identified a third type of repeat element called *Streptococcus pneumoniae* Rho-independent Terminator-like Element (SPITE). Like the previously known BOX and RUPS repeat elements, SPRITE is thought to contribute to genome evolution of the pneumococcus and is important in termination of transcription (Croucher et al., 2011b). Another factor that contributes to the high *S. pneumoniae* recombination is the fact that the pan-genome of the organism is open which means that the pan-genome has an infinite size and thus provides a rapid response to diverse environments (Donati et al., 2010). Phylogenetic studies on *S. pneumoniae* have shown that, the high rates of recombination can result in the elimination of any deep-rooted phylogenetic signal (Feil and Spratt, 2001). However, this does not prevent the formation of distinct pneumococcal lineages or clones but makes such clones relatively unstable compared to several other bacteria (Feil et al., 2003; Donati et al., 2010).

**Figure 1:**
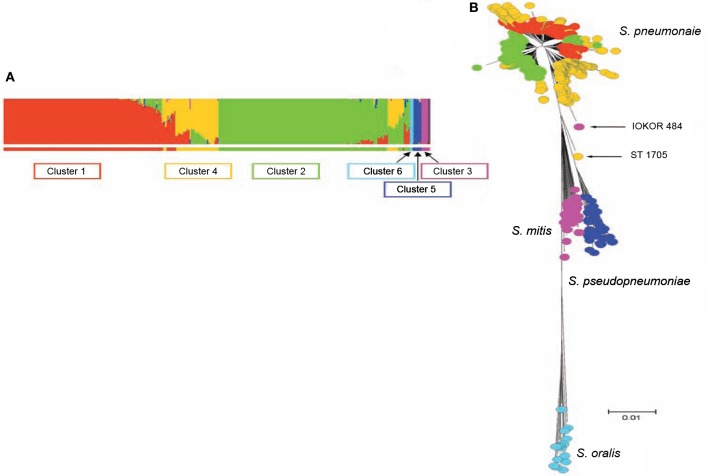
**(A)** Admixture analysis of 2024 distinct streptococcal genotypes of *S. pneumoniae, S. oralis, S. mitis, and S. pseudopneumoniae* based on Bayesian Analysis of Population Structure. **(B)** Minimum evolution tree constructed from concatenates of the different streptococci species. The streptococci fell into six clusters shown by the different colors (coloring of taxa in **B** corresponds to colors of BAPS clusters in **A**). Three BAPS clusters (including Clusters 1, 2, and 4) were associated with *S. pneumoniae* subpopulations, while the other three BAPS clusters namely, clusters 3, 5, and 6 were associated with *S. mitis, S. pseudopneumoniae, and S. oralis*, respectively. Relatively, *S. pneumoniae* cluster 4 appears heterogeneous and the isolates of this BAPS cluster are composed of a high proportion of mosaics due to genetic material from other clusters of *S. pneumoniae* and other streptococci. Adapted from Hanage et al. (2009).

*S. pneumoniae* interspecies recombination is usually related to genetic exchange with streptococci of the mitis group (Hanage et al., 2009). The mitis group of streptococci currently includes *S. pneumoniae* and 10 other members: *S. oralis*, *S. mitis*, *S. infantis*, *S. sanguis*, *S. gordonii*, *S. pseudopneumoniae, S. cristatus*, *S. oligofermentans*, *S. parasanguinis*, and *S. peroris*. *S. pneumoniae* which is the main pathogen in the group is closely related to *S. oralis*, and both species are believed to have evolved from a common ancestor (Kilian et al., 2008). Genetic exchange between *S. pneumoniae* and other mitis streptococci is facilitated by the co-habitation of these organisms in the nasopharynx, as well as the natural transformability of *S. pneumoniae* (Kilian et al., 2008). Based on sequence analysis of *pbp* genes, *S. mitis* and *S. oralis* have been found to donors of chromosomal DNA to *S. pneumoniae* in the evolution of mosaic penicillin-binding protein genes (Zerfass et al., 2009). Such mosaic pneumococcal genes from homologous recombination with the mitis group streptococci have also been observed in virulence genes such as *lytA, nanA, pspA, and pspC* (Johnston et al., 2010). Using MLST data, Hanage et al. (2009) investigated recombination among *S. pneumoniae* and other mitis group streptococci, and observed a distinct population of *S. pneumoniae* that exhibit hyper-recombination (Figure 1). This pneumococcal sub-population which was the main recipient of genetic material from other mitis group streptococci showed significantly higher levels of resistance for various antibiotics (penicillin, erythromycin, tetracycline, chloramphenicol, and cefotaxime) compared to other pneumococcal sub-populations which did not show evidence of recombination. While the basis of such a pneumococcal population exhibiting hyper-recombination is poorly understood, in terms of genome evolution, it is possible that the population may possess extraordinarily high density of repeat elements (Hoskins et al., 2001; Tettelin et al., 2001). Defects in the DNA mismatch repair system may also contribute to the hyper-recombination of this pneumococcal sub-population (Denamur and Matic, 2006; Hall and Henderson-Begg, 2006; Henderson-Begg et al., 2010). Very recently, Croucher et al. (2012) have demonstrated that the location and selective advantage of accessory genome loci may have the greatest mechanistic impact on homologous recombination occurring between lineages of the pneumococcal species. While there is a distinct pneumococcal population that exhibit hyper-recombination, there is no evidence of a pneumococcal population that exhibit hyper-mutation (Henderson-Begg et al., 2010).

Pneumococcal evolutionary events frequently occur at the capsular locus (*cps*), which encodes the pneumococcal capsule. The pneumococcal *cps* genes are flanked by the conserved *dexB* and *aliA* genes and synthesize capsule polysaccharide via the Wzx/Wzy-dependent pathway using the regulatory and processing genes *wzg*, *wzh*, *wzd*, and *wze* (Kolkman et al., 1998; Garcia et al., 2000). The only exceptions are serotypes 3 and 37 which utilize a synthase pathway for capsular biosynthesis (Cartee et al., 2001; Llull et al., 2001). Homologous recombination occurs at the flanking regions common among different serotypes. Each pneumococcal *cps* has a serotype specific region, where recombinational events lead to capsular switching and the possible formation of vaccine escape variants (Golubchik et al., 2012). Additionally, capsular recombinational events resulting in some pneumococcal capsular types may be associated with increase in virulence (Hu et al., 2012). It appears that some capsular genes undergo recombinational exchange among pneumococcal lineages more commonly than others. Several reasons may account for this observation, including differences in the genetic organization within the capsular loci (Bentley et al., 2006), the capabilities of the pneumococcal isolates to be transformed (Chen and Dubnau, 2004), and co-existence of isolates of different pneumococcal serotypes in the nasophyarnx. Within the pneumococcal *cps* loci, several mutational events have also been documented (Bentley et al., 2006), with some recent data suggesting that mutational events are also important in capsular switching (Sheppard et al., 2010).

## Conclusions

Transmission of the pneumococcus is a highly efficient process that usually occurs through respiratory droplets from asymptomatic carriers. Following acquisition, the pneumococcus may only establish in the nasopharynx of the new host, or further progress to sites such as the lungs and cause disease. Pneumococcus transmission risk factors, as well as factors involved in its translocation from the nasophyarnx to diseases sites are still not fully understood. Pneumococcal evolution is dominated by recombination which may be attributed to the relatively high density of repeat elements in the genome and also the fact that the pan-genome of the organism is open. The recombinational events usually involve genetic exchange with streptococci of the mitis group and some pneumococci are thought to exhibit hyper-recombination.

### Conflict of interest statement

The author declares that the research was conducted in the absence of any commercial or financial relationships that could be construed as a potential conflict of interest.

## References

[B1] Aniasson G. Alm B. Andersson B. Larsson P. Nylén O. Peterson H. (1992). Nasopharyngeal colonization during the first years of life. J. Infect. Dis. 165, 38–42 10.1093/infdis/165-supplement_1-s38 1588174

[B2] Arai J. Hotomi M. Hollingshead S. K. Ueno Y. Briles D. E. Yamanaka N. (2011). *Streptococcus pneumoniae* isolates from middle ear fluid and nasopharynx of children with acute otitis media exhibit phase variation. J. Clin. Microbiol. 49, 1646–1649 10.1128/JCM.01990-10 21346044 PMC3122802

[B3] Aras R. Kang J. Tschumi A. I. Harasaki Y. Blaser M. J. (2003). Extensive repetitive DNA facilitates prokaryotic genome plasticity. Proc. Natl. Acad. Sci. U.S.A. 100, 13579–13584 10.1073/pnas.1735481100 14593200 PMC263856

[B4] Bentley S. D. Aanensen D. M. Mavroidi A. Saunders D. Rabbinowitsch E. Collins M. (2006). Genetic analysis of the capsular biosynthetic locus from all 90 pneumococcal serotypes. PLoS Genet. 2:e31 10.1371/journal.pgen.0020031 16532061 PMC1391919

[B6] Bogaert D. De Groot R. Hermans P. W. (2004). *Streptococcus pneumoniae* colonisation: the key to pneumococcal disease. Lancet Infect. Dis. 4, 144–154 10.1016/S1473-3099(04)00938-7 14998500

[B7] Bohr V. Paulson O. B. Rasmussen N. (1984). Pneumococcal meningitis. Late neurologic sequelae and features of prognostic impact. Arch. Neurol. 41, 1045–1049 10.1001/archneur.1984.04050210043012 6477211

[B8] Brugger S. D. Frey P. Aebi S. Hinds J. Muhlemann K. (2010). Multiple colonization with *S. pneumoniae* before and after introduction of the seven-valent conjugated pneumococcal polysaccharide vaccine. PLoS ONE 5:e11638 10.1371/journal.pone.0011638 20661289 PMC2905437

[B9] Cartee R. T. Forsee W. T. Jensen J. W. Yother J. (2001). Expression of the *Streptococcus pneumoniae* type 3 synthase in *Escherichia coli*. Assembly of type 3 polysaccharide on a lipid primer. J. Biol. Chem. 276, 48831–48839 10.1074/jbc.M106481200 11684683

[B10] Chen I. Dubnau D. (2004). DNA uptake during bacterial transformation. Nat. Rev. Microbiol. 2, 241–249 10.1038/nrmicro844 15083159

[B11a] Croucher N. J. Harris S. R. Barquist L. Parkhill J. Bentley S. D. (2012). A high-resolution view of genome-wide pneumococcal transformation. PLoS Pathog. 8:e1002745 10.1371/journal.ppat.1002745 22719250 PMC3375284

[B11] Croucher N. J. Harris S. R. Fraser C. Quail M. A. Burton J. Van Der Linden M. (2011a). Rapid pneumococcal evolution in response to clinical interventions. Science 331, 430–434 10.1126/science.1198545 21273480 PMC3648787

[B12] Croucher N. J. Vernikos G. S. Parkhill J. Bentley S. D. (2011b). Identification, variation and transcription of pneumococcal repeat sequences. BMC Genome 12:120 10.1186/1471-2164-12-120 21333003 PMC3049150

[B13] Denamur E. Matic I. (2006). Evolution of mutation rates in bacteria. Mol. Microbiol. 60, 820–827 10.1111/j.1365-2958.2006.05150.x 16677295

[B14] Donati C. Hiller N. L. Tettelin H. Muzzi A. Croucher N. J. Angiuoli S. V. (2010). Structure and dynamics of the pan-genome of *Streptococcus pneumoniae* and closely related species. Genome Biol. 11:R107 10.1186/gb-2010-11-10-r107 21034474 PMC3218663

[B15] Feil E. J. Cooper J. E. Grundmann H. Robinson D. A. Enright M. C. Berendt A. (2003). How clonal is *Staphylococcus aureus*? J. Bacteriol. 185, 3307–3316 10.1128/JB.185.11.3307-3316.2003 12754228 PMC155367

[B16] Feil E. J. Smith J. M. Enright M. C. Spratt B. G. (2000). Estimating recombinational parameters in *Streptococcus pneumoniae* from multilocus sequence typing data. Genetics 154, 1439–1450 10747043 10.1093/genetics/154.4.1439 PMC1461021

[B17] Feil E. J. Spratt B. G. (2001). Recombination and the population structures of bacterial pathogens. Annu. Rev. Microbiol. 55, 561–590 10.1146/annurev.micro.55.1.561 11544367

[B18] Frederiksen B. Henrichsen J. (1988). Throat carriage of *Streptococcus pneumoniae* and *Streptococcus pyogenes* among infants and children in Zambia. J. Trop. Pediatr. 34, 114–117 10.1093/tropej/34.3.114 3043011

[B19] Garcia E. Llull D. Munoz R. Mollerach M. Lopez R. (2000). Current trends in capsular polysaccharide biosynthesis of *Streptococcus pneumoniae*. Res. Microbiol. 151, 429–435 10.1016/S0923-2508(00)00173-X 10961455

[B20] Givon-Lavi N. Fraser D. Porat N. Dagan R. (2002). Spread of *Streptococcus pneumoniae* and antibiotic-resistant *S. pneumoniae* from day-care center attendees to their younger siblings. J. Infect. Dis. 186, 1608–1614 10.1086/345556 12447737

[B21] Goldblatt D. Hussain M. Andrews N. Ashton L. Virta C. Melegaro A. (2005). Antibody responses to nasopharyngeal carriage of *Streptococcus pneumoniae* in adults: a longitudinal household study. J. Infect. Dis. 192, 387–393 10.1086/431524 15995951

[B22] Golubchik T. Brueggemann A. B. Street T. Gertz R. E. Jr. Spencer C. C. Giannoulatou E. (2012). Pneumococcal genome sequencing tracks a vaccine escape variant formed through a multi-fragment recombination event. Nat. Genet. 44, 352–355 10.1038/ng.1072 22286217 PMC3303117

[B23] Granat S. M. Mia Z. Ollgren J. Herva E. Das M. Piirainen L. (2007). Longitudinal study on pneumococcal carriage during the first year of life in Bangladesh. Pediatr. Infect. Dis. J. 26, 319–324 10.1097/01.inf.0000257425.24492.11 17414395

[B24] Gratten M. Gratten H. Poli A. Carrad E. Raymer M. Koki G. (1986). Colonisation of *Haemophilus influenzae* and *Streptococcus pneumoniae* in the upper respiratory tract of neonates in Papua New Guinea: primary acquisition, duration of carriage, and relationship to carriage in mothers. Biol. Neonate 50, 114–120 3489488 10.1159/000242576

[B25] Gratten M. Manning K. Dixon J. Morey F. Torzillo P. Hanna J. (1994). Upper airway carriage by *Haemophilus influenzae* and *Streptococcus pneumoniae* in Australian aboriginal children hospitalised with acute lower respiratory infection. Southeast Asian J. Trop. Med. Public Health 25, 123–131 7825002

[B26] Gray B. M. Converse G. M. Dillon H. C. (1980). Epidemiologic studies of *Streptococcus pneumoniae* in infants: acquisition, carriage, and infection during the first 24 months of life. J. Infect. Dis. 142, 923–933 10.1093/infdis/142.6.923 7462701

[B27] Hall L. M. C. Henderson-Begg S. K. (2006). Hypermutable bacteria isolated from humans – a critical analysis. Microbiology 152, 2505–2514 10.1099/mic.0.29079-0 16946246

[B28] Hanage W. P. Fraser C. Tang J. Connor T. R. Corander J. (2009). Hyper-recombination, diversity, and antibiotic resistance in pneumococcus. Science 324, 1454–1457 10.1126/science.1171908 19520963

[B29] Harputluoglu U. Egeli E. Sahin I. Oghan F. Ozturk O. (2005). Nasopharyngeal aerobic bacterial flora and *Staphylococcus aureus* nasal carriage in deaf children. Int. J. Pediatr. Otorhinolaryngol. 69, 69–74 10.1016/j.ijporl.2004.08.005 15627450

[B30] Henderson-Begg S. K. Sheppard C. L. George R. C. Livermore D. M. Hall L. M. (2010). Mutation frequency in antibiotic-resistant and -susceptible isolates of *Streptococcus pneumoniae*. Int. J. Antimicrob. Agents 35, 342–346 10.1016/j.ijantimicag.2009.12.015 20149603

[B31] Hoskins J. Alborn W. E. Arnold J. Blaszczak L. C. Burgett S. Dehoff B. S. (2001). Genome of the bacterium *Streptococcus pneumoniae* strain R6. J. Bacteriol. 183, 5709–5717 10.1128/JB.183.19.5709-5717.2001 11544234 PMC95463

[B32] Hu F. Z. Eutsey R. Ahmed A. Frazao N. Powell E. Hiller N. L. (2012). *In vivo* capsular switch in *Streptococcus pneumoniae*- analysis by whole genome sequencing. PLoS ONE 7:e47983 10.1371/journal.pone.0047983 23144841 PMC3493582

[B33] Hussain M. Melegaro A. Pebody R. G. George R. Edmunds W. J. Talukdar R. (2005). A longitudinal household study of *Streptococcus pneumoniae* nasopharyngeal carriage in a UK setting. Epidemiol. Infect. 133, 891–898 10.1017/S0950268805004012 16181510 PMC2870321

[B34] Johnston C. Hinds J. Smith A. Van Der Linden M. Van Eldere J. Mitchell T. J. (2010). Detection of large numbers of pneumococcal virulence genes in streptococci of the mitis group. J. Clin. Microbiol. 48, 2762–2769 10.1128/JCM.01746-09 20519466 PMC2916619

[B35] Kilian M. Poulsen K. Blomqvist T. Håvarstein L. S. Bek-Thomsen M. Tettelin H. (2008). Evolution of *Streptococcus pneumoniae* and its close commensal relatives. PLoS ONE 3:e2683 10.1371/journal.pone.0002683 18628950 PMC2444020

[B36] Kolkman M. A. Van Der Zeijst B. A. Nuijten P. J. (1998). Diversity of capsular polysaccharide synthesis gene clusters in *Streptococcus pneumoniae*. J. Biochem. (Tokyo) 123, 937–945 9562629 10.1093/oxfordjournals.jbchem.a022028

[B37] Labout J. A. M. Duijts L. Arends L. R. Jaddoe V. W. V. Hofman A. De Groot R. (2008). Factors associated with pneumococcal carriage in healthy Dutch infants: the generation R study. J. Pediatr. 153, 771–776 10.1016/j.jpeds.2008.05.061 18621390

[B38] Lankinen K. S. Leinonen M. Tupasi T. E. Haikala R. Ruutu P. (1994). Pneumococci in nasopharyngeal samples from Filipino children with acute respiratory infections. J. Clin. Microbiol. 32, 2948–2952 7883882 10.1128/jcm.32.12.2948-2952.1994 PMC264205

[B39] Leimkugel J. Adams Forgor A. Gagneux S. Pfluger V. Flierl C. Awine E. (2005). An outbreak of serotype 1 *Streptococcus pneumoniae* meningitis in northern Ghana with features that are characteristic of *Neisseria meningitidis* meningitis epidemics. J. Infect. Dis. 192, 192–199 10.1086/431151 15962213

[B40] Libson S. Dagan R. Greenberg D. Porat N. Trepler R. Leiberman A. (2005). Nasopharyngeal carriage of *Streptococcus pneumoniae* at the completion of successful antibiotic treatment of acute otitis media predisposes to early clinical recurrence. J. Infect. Dis. 191, 1869–1875 10.1086/429918 15871120

[B41] Lloyd-Evans N. O'Dempsey T. J. Baldeh I. Secka O. Demba E. Todd J. E. (1996). Nasopharyngeal carriage of pneumococci in Gambian children and in their families. Pediatr. Infect. Dis. J. 15, 866–871 8895917 10.1097/00006454-199610000-00007

[B42] Llull D. Garcia E. Lopez R. (2001). Tts, a processive β-glucosyltransferase of *Streptococcus pneumoniae* directs the synthesis of the branched type 37 capsular polysaccharide in pneumococcus and other gram-positive species. J. Biol. Chem. 276, 21053–21061 10.1074/jbc.M010287200 11264282

[B43] Mastro T. D. Nomani N. K. Ishaq Z. Ghafoor A. Shaukat N. F. Esko E. (1993). Use of nasopharyngeal isolates of *Streptococcus pneumoniae* and *Haemophilus influenzae* from children in Pakistan for surveillance for antimicrobial resistance. Pediatr. Infect. Dis. J. 12, 824–830 8284119 10.1097/00006454-199310000-00006

[B44] Mrozek-Budzyn D. (2010). The significance of epidemiological studies for progress of measles elimination. Przegl. Epidemiol. 64, 361–366 20976947

[B45] Musher D. M. (2004). A pathogenic categorization of clinical syndromes caused by *Streptococcus pneumoniae*, in The Pneumococcus, ed Tuomanen E. I. (Washington, DC: ASM Press), 211–220

[B46] Obaro S. Adegbola R. (2002). The pneumococcus: carriage, disease and conjugate vaccines. J. Med. Microbiol. 51, 98–104 11863272 10.1099/0022-1317-51-2-98

[B47] Obaro S. K. Adegbola R. A. Banya W. A. Greenwood B. M. (1996). Carriage of pneumococci after pneumococcal vaccination. Lancet 348, 271–272 8684225 10.1016/s0140-6736(05)65585-7

[B48] O'Brien K. L. Wolfson L. J. Watt J. P. Henkle E. Deloria-Knoll M. (2009). Burden of disease caused by *Streptococcus pneumoniae* in children younger than 5 years: global estimates. Lancet 374, 893–902 10.1016/S0140-6736(09)61204-6 19748398

[B49] Ring A. Weiser J. N. Tuomanen E. I. (1998). Pneumococcal trafficking across the blood-brain barrier. Molecular analysis of a novel bidirectional pathway. J. Clin. Invest. 102, 347–360 10.1172/JCI2406 9664076 PMC508893

[B50a] Rosen C. Christensen P. Hovelius B. Prellner K. (1984). A longitudinal study of the nasopharyngeal carriage of pneumococci as related to pneumococcal vaccination in children attending day-care centres. Acta Otolaryngol 98, 524–532 6524348 10.3109/00016488409107593

[B50] Sheppard C. L. Pichon B. George R. C. Hall L. M. (2010). *Streptococcus pneumoniae* isolates expressing a capsule with epitopes of both serotypes 6A and 6B. Clin. Vaccine Immunol. 17, 1820–1822 10.1128/CVI.00335-10 20876824 PMC2976087

[B51] Sleeman K. L. Daniels L. Gardiner M. Griffiths D. Deeks J. J. Dagan R. (2005). Acquisition of *Streptococcus pneumoniae* and nonspecific morbidity in infants and their families: a cohort study. Pediatr. Infect. Dis. J. 24, 121–127 15702039 10.1097/01.inf.0000151030.10159.b1

[B52] Sleeman K. L. Griffiths D. Shackley F. Diggle L. Gupta S. Maiden M. C. (2008). Capsular serotype-specific attack rates and duration of carriage of *Streptococcus pneumoniae* among adlosescents. J. Med. Microbiol. 57, 185–189 10.1086/505710 16897668

[B53] Tettelin H. Nelson K. E. Paulsen I. T. Eisen J. A. Read T. D. Peterson S. (2001). Complete genome sequence of a virulent isolate of *Streptococcus pneumoniae*. Science 293, 498–506 10.1126/science.1061217 11463916

[B54] Tuomanen E. I. Austrian R. Masure H. R. (1995). Pathogenesis of pneumococcal infection. N. Engl. J. Med. 332, 1280–1284 10.1056/NEJM199505113321907 7708073

[B55] Weiser J. N. Austrian R. Sreenivasan P. K. Masure H. R. (1994). Phase variation in pneumococcal opacity: relationship between colonial morphology and nasopharyngeal colonization. Infect. Immun. 62, 2582–2589 8188381 10.1128/iai.62.6.2582-2589.1994 PMC186548

[B56] Zerfass I. Hakenbeck R. Denapaite D. (2009). An important site in PBP2x of penicillin-resistant clinical isolates of *Streptococcus pneumoniae*: mutational analysis of Thr338. Antimicrob. Agents Chemother. 53, 1107–1115 10.1128/AAC.01107-08 19075056 PMC2650557

